# Atypical dermal melanocytosis: a diagnostic clue in constitutional mismatch repair deficiency syndrome

**DOI:** 10.1111/bjd.15532

**Published:** 2017-09-28

**Authors:** S. Polubothu, R.H. Scott, P. Vabres, V.A. Kinsler

**Affiliations:** ^1^ Paediatric Dermatology Department Great Ormond Street Hospital for Children London U.K; ^2^ Genetics and Genomic Medicine UCL Great Ormond Street Institute of Child Health UCL London U.K; ^3^ Clinical Genetics Department Great Ormond Street Hospital for Children London U.K; ^4^ Equipe d'Accueil 4271 Génétique des Anomalies du Développement Université de Bourgogne Franche‐Comté Dijon France; ^5^ Dermatology Centre Hospitalier Universitaire de Dijon Bourgogne Dijon France

dear editor, A 23‐month‐old female presented with abnormal cutaneous pigmentation since birth. She had a past history of mediastinal non‐Hodgkin lymphoma diagnosed at 9 months of age and treated with surgery and chemotherapy. Family history revealed her grandfathers were brothers, and both died of colon cancer in their fifth decade. Her parents were both fit and well in their early 30s.

The patient had Fitzpatrick skin type IV, with 10 café au lait macules of up to 10 cm in diameter, and six hypomelanotic macules of 0·5–1 cm. In addition, she had extensive atypical dermal melanocytosis, with multiple blue‐grey macules predominantly on her back, of 1–10 cm, with an irregular outline (Fig. 1). The remainder of her physical examination was normal. A clinical diagnosis of constitutional mismatch repair deficiency syndrome (CMMRD) was made.

**Figure 1:**
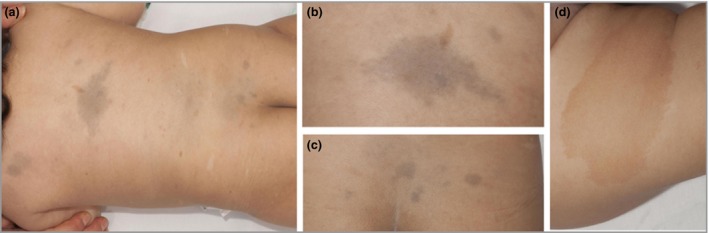
Dermal melanocytosis, hyperpigmentation and hypopigmentation in a 23‐month‐old female with constitutional mismatch repair deficiency syndrome (a). Blue‐grey macules of dermal melanocytosis were seen with irregular, ragged edges (b, c). Widespread hyperpigmented macules, again with ragged edges, were present with one large clearly defined café au lait macule (d).

Interphase fluorescence *in situ* hybridization revealed a normal germline female karyotype with no chromosomal rearrangements. Next‐generation sequencing (NGS) of leukocyte DNA of the mismatch repair genes *MLH1*,* MSH2* and *MSH6* revealed a novel homozygous mutation in *MSH6* (c.3934_3937dupGTTA, p.(Ile1313SerfsTer7)) predicted to be pathogenic, confirming the clinical diagnosis. Both parents were confirmed to be heterozygous for the same mutation. As somatic mutations in *GNA11* and *GNAQ* can be a cause of extensive or atypical dermal melanocytosis, DNA was extracted directly from a skin biopsy of an area of dermal melanocytosis. Targeted NGS of mutation hotspots (codons 183 and 209) revealed wild‐type sequence, excluding these as a cause of this aspect of the phenotype.

Mismatch repair is a critical mechanism during DNA replication for maintenance of genome integrity. Mutations in mismatch repair (MMR) genes *MLH1*,* MSH2*,* MSH6* or *PMS2* result in increased cancer susceptibility. Lynch syndrome, or hereditary nonpolyposis colorectal cancer, results from monoallelic (heterozygous) germline mutations in MMR genes. This autosomal dominant cancer predisposition syndrome is characterized by colorectal, endometrial, ovarian, gastric, small intestinal or urinary tract cancers from early adulthood. Tumorigenesis results from loss of the remaining MMR gene wild‐type alleles and accumulation of further mutations in cancer genes.

CMMRD is a rare autosomal recessive disease resulting from biallelic germline mutations, either homozygous or compound heterozygous, in one of the four mismatch repair genes. It can also be known as biallelic mismatch repair disorder (BMMRD), highlighting that a history of consanguinity is an important early diagnostic clue,^1^ as there may not be a striking family history of cancers in the extended family.^1^ It was first reported in 1999, and over 100 cases have been reported to date.^2^ In contrast to Lynch syndrome, which presents with cancers from the fourth decade, patients with biallelic MMR gene mutations develop a diverse spectrum of childhood malignancies, and often multiple malignancies, in early life. Central nervous system (CNS), haematological and colorectal malignancies predominate.^3^, ^4^ In the first 15 years of life, CNS tumours are the most frequently observed malignancies, the majority being high‐grade gliomas.^4^ Haematological malignancies are also frequently seen, of which the most common is non‐Hodgkin lymphoma. Non‐Hodgkin lymphoma and acute leukaemia are the earliest observed malignancies in CMMRD, presenting as early as 4 months of age.^4^

The skin offers valuable diagnostic clues in many DNA repair disorders. Café au lait macules are a recognized feature of CMMRD frequently causing diagnostic confusion with neurofibromatosis (NF)1. Unlike the café au lait macules observed in NF1, which have clearly defined borders, hyperpigmented macules in CMMRD often have ragged borders. Wimmer *et al*., in the largest comprehensive review of all published cases of CMMRD, described hyperpigmentation in 91 of 146 cases.^4^ Hypopigmentation was observed in nine of 146 patients. Other cutaneous features include so‐called ‘haemangiomas’ without further description reported in two cases^5^, ^6^ and sebaceous cysts in one.^7^

We reviewed all 43 published cases of biallelic *MSH6* mutations with regard to their cutaneous phenotype (Table S1; see Supporting Information). Hyperpigmentation was observed in 39 of 40 patients, and axillary freckling in four of 40; therefore patients can sometimes meet the diagnostic criteria for NF1. Hypopigmentation was reported in six of 40 patients; however, this may be due to under‐reporting as in the authors' experience this is a near universal finding in clinical practice.

Extensive or a typical dermal melanocytosis, as seen in this case, has not yet been described in CMMRD. However, examination of clinical images from other published case reports revealed dermal melanocytosis in a further case.^8^ Additionally, in 1998, prior to the first description of CMMRD, one of the authors reported a case of suspected NF1 in a child with atypical dermal melanocytosis, ocular melanosis and café au lait macules, who had developed non‐Hodgkin lymphoma. In hindsight we believe that a diagnosis of CMMRD is likely.^9^ Dermal melanocytosis is common in the general population in the form of Mongolian blue spots, and has been shown to be under‐reported by clinicians.^10^ We hypothesize that it may be an under‐reported feature in CMMRD.

This case highlights the early diagnostic clues offered by thorough cutaneous examination in CMMRD. Clinicians should be alert to the diverse range of pigmentary abnormalities observed in CMMRD, so as to prompt consideration of this diagnosis in cases presenting with dyspigmentation with an appropriate family or personal history of malignancy or a history of consanguinity. Early diagnosis can then lead to the implementation of screening for malignancies in both the proband and the wider family.^1^

## Supporting information


**Table S1** Cutaneous features observed in all published cases of biallelic *MSH6* mutation.
