# Supplementary material for: Atypical dermal melanocytosis: a diagnostic clue in constitutional mismatch repair deficiency syndrome
Source: Br J Dermatol. 2017 Sep 28;177(5):e185–6. doi: 10.1111/bjd.15532 (PMC5725661; doi:10.1111/bjd.15532)
Supplement: Supplementary file 1 — Table S1 Cutaneous features observed in all published cases of biallelic MSH6 mutation. [file BJD-177-e185-s001.docx]

**Supporting Information**

**Table S1: Cutaneous features observed in all published cases of biallelic *MSH6* mutation**

| **Reference** | **Family**  **(Case)** | ***MSH6***  **Mutation** | **Malignancy** | **Age at diagnosis of malignancy**  **(years)** | **Cutaneous Features** |
| --- | --- | --- | --- | --- | --- |
| Menko et al., 2004 | 1 (1) | Homozygous  c.3386_3388delGTG | - Oligodendroglioma - Rectosigmoid Carcinoma | 10  12 | - Café-au-lait macules |
| Hegde et al., 2005 | 2 (1) | Homozygous  c.3634insT | - Lymphoblastic lymphoma - Colonic Carcinoma | 5  8 | - Café-au-lait macules - Axillary freckling |
| Hegde et al., 2005 | 2 (2) | Homozygous  c.3634insT | - Glioblastoma Multiforme | 8 | - Café-au-lait macules |
| Ostergaard et al., 2005 | 3 (1) | Compound Heterozygous  c.3073G>A  c.3609_3612del | - Pilocytic Astrocytoma - Anaplastic Astrocytoma - T-cell Lymphoma | 9  9  10 | - Café-au-lait macules - Left axillary freckling |
| Ostergaard et al., 2005 | 3 (2) | Compound Heterozygous  c.3073G>A  c.3609_3612del | - Spinal Glioblastoma | 2 | - Café-au-lait macules - Axillary freckling |
| Plaschke et al., 2006 | 4 (1) | Compound Heterozygous  c.3991C>T  c3226C>T | - Rectal Carcinoma - Endometrial Carcinoma | 19  24 | - Café-au-lait macules |
| Plaschke et al., 2006 | 5 (1) | Compound Heterozygous  p.C765W  p.V878A  (nucleotide change not specified) | - Colonic Carcinoma | 31 | - Not stated |
| Scott et al., 2007 | 6 (1) | Compound Heterozygous  c.642C>G  c.458-1G>A | - Medulloblastoma - Acute Myeloid Leukemia - Colonic Carcinomas | 7  10  13 | - Hyperpigmented - Hypopigmented - Sebaceous cysts |
| Etzler et al., 2008 | 7 (1) | Homozygous  c.4002 ̷ 31_4002 ̷ 8delinsTTAAAAAAAAAAAAAAAAAAGTTT | - Medulloblastoma - Acute Myeloid Leukaemia | 6  9 | - Café-au-lait macules |
| Etzler et al., 2008 | 7 (2) | Homozygous  c.4002 ̷ 31_4002 ̷ 8delinsTTAAAAAAAAAAAAAAAAAAGTTT | - Glioblastoma multiforme | 9 | - Café-au-lait macules - Hypopigmented spots |
| Peters et al., 2009 | 8 (1) | Compound Heterozygous  c.1634-1635delAA  c.3957-3958insTCAAAAGGGACATAGAAAA | - T-cell Lymphoblastic lymphoma | 8 | - Segmental café-au-lait macules |
| Ripperger et al., 2010 | 9 (1) | Homozygous  c.691delG | - Non-Hodgkins Lymphoma - Colorectal cancer | 6  13 | - Café-au-lait macules - Lateral conjunctival melanosis of left eye |
| Ilencikova et al., 2011 | 10 (1) | Homozygous  c.3261insC | - Astrocytoma - T-cell Non-Hodkins Lymphoma | 11  12 | - Café-au-lait macules |
| Ilencikova et al., 2011 | 10 (2) | Homozygous  c.3261insC | - Gliobastoma | 10 | - Café-au-lait macules |
| Ilencikova et al., 2011 | 10 (3) | Homozygous  c.3261insC | - None yet at 1 year |  | - Café-au-lait macules |
| Bakry et al., 2014 | 11 (1) | Homozygous  c.3633insT | - Glioblastoma multiforme - T-cell Lymphoma - Gastrointestinal adenomas | 8  10  12 | - Café-au-lait macules |
| Bakry et al., 2014 | 12 (1) | Homozygous  c.3417delC | - Anaplastic Oligodindrioglioma | 10 | - Café-au-lait macules |
| Bakry et al., 2014 | 13 (1) | Homozygous  c.3946G>A | - Anaplastic Astroctyoma | 11 | - Café-au-lait macules |
| Bakry et al., 2014 | 14 (1) | Compound Heterozygous  c.3984-3987dupGTCA  c.3959delCAAG | - Gastrointestinal adenoma | 11 | - Café-au-lait macules |
| Bakry et al., 2014 | 14 (2) | Compound Heterozygous  c.3984-3987dupGTCA  c.3959delCAAG | - T-cell Lymphoma - Gastrointestinal adenoma | 6  9 | - Café-au-lait macules - Freckles |
| Bakry et al., 2014 | 14 (3) | Compound Heterozygous  c.3984-3987dupGTCA  c.3959delCAAG | - Glioblastoma Multiforme | 12 | - Café-au-lait macules |
| Bougeard et al., 2014 | 15 (1) | Compound Heterozygous  c.3261dup  c.2561_2563del | - Caecal adenocarcinoma - Sigmoid colon adenocarcinoma - Ureteric transitional cell carcinoma | 17  20  25 | - Café-au-lait macules |
| Bougeard et al., 2014 | 15 (2) | Presumed Compound Heterozygous | - Glioblastoma | 5 | - Not stated |
| Hoell et al., 2014 | 16 (1) | Homozygous  29bp deletion | - T-cell non-Hodgkin lymphoma - Colonic adenocarcinoma | 1  12 | - Not stated |
| Rahner et al., 2014 | 17 (1) | Compound Heterozygous  c.1806_1809delAAAG  c.3226C>T | - Colonic Adenocarcinoma | 17 | - Vitiligo - SLE (16 years) |
| Baris et al., 2014 | 18 (1) | Compound Heterozygous  c.3984_3987dupGTCA  c.3959_3962delCAAG | - T-cell lymphoma - Colonic adenoma | 6  11 | - Café-au-lait macules |
| Baris et al., 2014 | 18(2) | Compound Heterozygous  c.3984_3987dupGTCA  c.3959_3962delCAAG | - Duodenal adenoma - Glioblastoma multiforme | 9.5  10.5 | - Café-au-lait macules |
| Baris et al., 2014 | 18 (3) | Compound Heterozygous  c.3984_3987dupGTCA  c.3959_3962delCAAG | - Duodenal adenoma | 11 | - Café-au-lait macules |
| Baris et al., 2014 | 19 (1) | Homozygous  c.3603_3603delAGTG | - Glioblastoma Multiforme | 2 | - Café-au-lait macules |
| Baris et al., 2014 | 19 (2) | Homozygous  c.3603_3603delAGTG | - No malignancy at 6 years |  | - Café-au-lait macules |
| Baris et al., 2014 | 19 (3) | Homozygous  c.3603_3603delAGTG | - Medulloblastoma - Acute myeloid leukaemia | 6  8 | - Café-au-lait macules |
| Baris et al., 2014 | 19 (4) | Homozygous  c.3603_3603delAGTG | - Medulloblastoma | 5 | - Café-au-lait macules |
| Baris et al., 2014 | 19 (5) | Homozygous  c.3603_3603delAGTG | - No malignancy at 4 years |  | - Café-au-lait macules |
| Auclair et al., 2014 | 20 (1) | Compound Heterozygous  c.1596_1597dupT  c.3261delC | - Glioblastoma | 7 years | - Café-au-lait macules |
| Auclair et al., 2014 | 20 (2) | Compound Heterozygous  c.1596_1597dupT  c.3261delC | - Multiple adenomatous polyps high degree of dysplasia | 9 years | - Café-au-lait macules - Lisch nodules |
| Elhasid et al., 2015 | 21 (1) | Homozygous c.2314C>T | - Acute Myeloid Leukaemia | 2 | - Café-au-lait macules - Hypopigmented spots |
| Elhasid et al., 2015 | 21 (2) | Homozygous c.2314C>T | - None at 5 years |  | - Café-au-lait macules - Hypopigmented spots |
| Lavoine et al., 2015 | 22 (1) | Homozygous  c.2216C>A | - Glioblastoma | 6 | - Café-au-lait macules |
| Lavoine et al., 2015 | 22 (2) | Homozygous  c.2216C>A | - Astrocytoma | 9 | - Café-au-lait macules |
| Lavoine et al., 2015 | 23 (1) | Homozygous  c.3991C>T | - Colorectal Cancer | 11 | - Café-au-lait macules |
| Lavoine et al., 2015 | 24 (1) | Compound Heterozygous  c.3261dup  c.2561_2563del | - Adenoma - Colorectal cancer - Colorectal cancer - Urinary Tract Carcinoma | 14  17  19  24 | - Café-au-lait macules |
| Lavoine et al., 2015 | 25 (1) | Compound Heterozygous  c.1596_1597dup  c.3261del | - Glioblastoma | 7 | - Café-au-lait macules |
| Lavoine et al., 2015 | 26 (1) | Homozygous  c.1763_1771dup | - T-cell Lymphoblastic Lymphoma - T-cell Lymphoblastic Lymphoma - Glioblastoma - Colorectal Cancer | 6  11  14  14 | - Café-au-lait macules - Hypopigmented spots |

**References**

1. Menko FH, Kaspers GL, Meijer GA, Claes K, van Hagen JM, Gille JJ. A homozygous MSH6 mutation in a child with cafe-au-lait spots, oligodendroglioma and rectal cancer. Fam Cancer. 2004;3(2):123-7.

2. Hegde MR, Chong B, Blazo ME, Chin LH, Ward PA, Chintagumpala MM, et al. A homozygous mutation in MSH6 causes Turcot syndrome. Clinical cancer research : an official journal of the American Association for Cancer Research. 2005;11(13):4689-93.

3. Ostergaard JR, Sunde L, Okkels H. Neurofibromatosis von Recklinghausen type I phenotype and early onset of cancers in siblings compound heterozygous for mutations in MSH6. American journal of medical genetics Part A. 2005;139a(2):96-105; discussion 96.

4. Plaschke J, Linnebacher M, Kloor M, Gebert J, Cremer FW, Tinschert S, et al. Compound heterozygosity for two MSH6 mutations in a patient with early onset of HNPCC-associated cancers, but without hematological malignancy and brain tumor. Eur J Hum Genet. 2006;14(5):561-6.

5. Scott RH, Mansour S, Pritchard-Jones K, Kumar D, MacSweeney F, Rahman N. Medulloblastoma, acute myelocytic leukemia and colonic carcinomas in a child with biallelic MSH6 mutations. Nature clinical practice Oncology. 2007;4(2):130-4.

6. Etzler J, Peyrl A, Zatkova A, Schildhaus HU, Ficek A, Merkelbach-Bruse S, et al. RNA-based mutation analysis identifies an unusual MSH6 splicing defect and circumvents PMS2 pseudogene interference. Human Mutation. 2008;29(2):299-305.

7. Peters A, Born H, Ettinger R, Levonian P, Jedele KB. Compound heterozygosity for MSH6 mutations in a pediatric lymphoma patient. Journal of Pediatric Hematology/Oncology. 2009;31(2):113-5.

8. Ripperger T, Beger C, Rahner N, Sykora KW, Bockmeyer CL, Lehmann U, et al. Constitutional mismatch repair deficiency and childhood leukemia/lymphoma – report on a novel biallelic MSH6 mutation. Haematologica. 2010;95(5):841-4.

9. Ilencikova D, Sejnova D, Jindrova J, Babal P. High-grade brain tumors in siblings with biallelic MSH6 mutations. Pediatric blood & cancer. 2011;57(6):1067-70.

10. Bakry D, Aronson M, Durno C, Rimawi H, Farah R, Alharbi QK, et al. Genetic and clinical determinants of constitutional mismatch repair deficiency syndrome: report from the constitutional mismatch repair deficiency consortium. European journal of cancer (Oxford, England : 1990). 2014;50(5):987-96.

11. Bougeard G, Olivier-Faivre L, Baert-Desurmont S, Tinat J, Martin C, Bouvignies E, et al. Diversity of the clinical presentation of the MMR gene biallelic mutations. Familial Cancer. 2014;13(1):131-5.

12. Hoell JI, Gombert M, Ginzel S, Loth S, Landgraf P, Kafer V, et al. Constitutional mismatch repair-deficiency and whole-exome sequencing as the means of the rapid detection of the causative MSH6 defect. Klinische Padiatrie. 2014;226(6-7):357-61.

13. Rahner N, Hoefler G, Hogenauer C, Lackner C, Steinke V, Sengteller M, et al. Compound heterozygosity for two MSH6 mutations in a patient with early onset colorectal cancer, vitiligo and systemic lupus erythematosus. American journal of medical genetics Part A. 2008;146a(10):1314-9.

14. Baris HN, Barnes-Kedar I, Toledano H, Halpern M, Hershkovitz D, Lossos A, et al. Constitutional Mismatch Repair Deficiency in Israel: High Proportion of Founder Mutations in MMR Genes and Consanguinity. Pediatric blood & cancer. 2016;63(3):418-27.

15. Auclair J, Leroux D, Desseigne F, Lasset C, Saurin JC, Joly MO, et al. Novel biallelic mutations in MSH6 and PMS2 genes: gene conversion as a likely cause of PMS2 gene inactivation. Hum Mutat. 2007;28(11):1084-90.

16. Elhasid R, Dvir R, Keidar HR, Shachar SB, Bitan M, Solar I, et al. Management of acute myeloblastic leukemia in a child with biallelic mismatch repair deficiency. Journal of Pediatric Hematology/Oncology. 2015;37(8):e490-e3.

17. Lavoine N, Colas C, Muleris M, Bodo S, Duval A, Entz-Werle N, et al. Constitutional mismatch repair deficiency syndrome: Clinical description in a French cohort. Journal of medical genetics. 2015;52(11):770-8.
